# Ginkgetin aglycone ameliorates LPS-induced acute kidney injury by activating SIRT1 via inhibiting the NF-κB signaling pathway

**DOI:** 10.1186/s13578-017-0173-3

**Published:** 2017-08-23

**Authors:** Junwei Zhang, Suxia Yang, Fang Chen, Huicong Li, Baoping Chen

**Affiliations:** Department of Nephrology, Huaihe Hospital of Henan University, No. 115, Gulou District, Kaifeng, 475000 China

**Keywords:** Ginkgetin aglycone, Acute kidney injury, Inflammation, Apoptosis, LPS, SIRT1, NF-κB

## Abstract

**Background:**

Ginkgetin aglycone (GA), a novel *Ginkgo biloba* extract (GBE) by acid hydrolysis and recrystallization, is characterized by higher liposolubility and antioxidation than classical GBEs. There is no study depicting the functional role of GA in acute kidney injury (AKI). Here, we firstly reported the protective effect of GA on lipopolysaccharide (LPS)-induced AKI and its underlying mechanism.

**Methods:**

ELISA analysis was applied to measure plasma level of TNF-α and IL-6, and NF-κB activity in kidney homogenate. Renal function analysis was performed by detecting serum concentration of Kim-1 and urine level of BUN. Cell apoptosis in kidney tissues was detected by TUNEL assay and caspase-3 activity assay. qRT-PCR was conducted to determine mRNA expression of TNF-α, IL-6 and IκBα. Western blot was carried out to confirm expression of p-IκBα, SIRT1, and iNOS.

**Results:**

GA administration protected mice from LPS-induced AKI by attenuating inflammatory response, renal injury, as well as tubular apoptosis both in vivo. GA suppressed inflammatory response induced by LPS in HK-2 cells. Moreover, GA upregulated SIRT1 expression and blocked the NF-κB signaling pathway in LPS-induced AKT in vivo and vitro. Furthermore, suppression of SIRT1 abated the inhibitory effect of GA on LPS-induced inflammatory response and renal injury.

**Conclusions:**

GA prevented LPS-induced AKI by activating SIRT1 via inhibiting the NF-κB signaling pathway, providing new insights into the function and molecular mechanism of GA in AKI. Therefore, GA may be a promising therapeutic agent for the treatment of septic AKI.

## Background

Acute kidney injury (AKI), a systemic inflammatory response syndrome, is clinically characterized by severe tubular cell injury and rapid kidney dysfunction [1]. It is reported that AKI is associated with intrarenal and systemic inflammation, which leads to impaired urinary concentration and kidney injury [2]. It is increasingly recognized that severe sepsis and septic shock are the major causes of AKI in bacterium-infected intensive care unit (ICU) patients, accounting for approximately half of these AKI patients [3, 4]. Sepsis-induced AKI is increasingly considered as a global healthcare issue due to high incidence rates, along with considerable morbidity and mortality rates [5]. Unfortunately, despite significant advances in medicine and therapeutics, there is still no effective treatment available to prevent this devastating disease [6].

Lipopolysaccharide (LPS), a bacteria endotoxin which results in strong immune and inflammatory responses in animals, is reported to be involved in the pathogenesis of sepsis-induced AKI [7]. Therefore, LPS-induced AKI has been widely used as one of common animal models to elucidate the mechanisms underlying sepsis-induced AKI [8]. A growing body of evidence indicates that the inflammatory response inherent to sepsis is regarded as a direct mechanism of AKI [9]. During endotoxemia, LPS-induced toll-like receptor-4 (TLR-4) activation promotes expressions of inflammatory cytokines including tumor necrosis factor-α (TNF-α), interleukins (IL-1β and IL-6) through transcription of nuclear factor-κB (NF-κB), which is a crucial regulator of immune system [10]. The activated immune cells promote the production of reactive oxidative species (ROS), which is implicated in the pathogenesis of LPS-induced AKI [11, 12]. Sirtuin 1 (SIRT1), a member of NAD^+^-dependent class III histone deacetylases, is demonstrated to play an important role in the regulation of cellular oxidative stress response, metabolism, as well as inflammatory diseases including kidney disease by suppressing the release of pro-inflammatory cytokines [13]. It has been found that SIRT1 activation could ameliorate sepsis-induced inflammatory response and protect kidney from acute injury [14]. Recently, increasing studies have suggested that SIRT1 is a novel target to prevent kidney diseases [15, 16].

Extracts from the leaves of *Ginkgo biloba* have been widely used therapeutically in China and Western countries for centuries due to their antioxidant properties [17, 18]. Flavonoids and terpenoid, the most important active substances in standard *Ginkgo biloba* extract (GBE), possesses biological effects including free radical scavenging, antioxidant and anti-inflammatory activities [19]. Ginkgetin aglycone (GA), a novel GBE by acid hydrolysis and recrystallization, is characterized by higher liposolubility and antioxidation than classical GBEs [20]. Up to now, there is no report depicting the functional role of GA in AKI. The present study aimed to investigate the protective effect of GA against LPS-induced AKI and its underlying mechanism to provide more rationality for its application in the treatment of AKI.

## Methods

### Reagents

GA (purity ≥ 98%) was purchased from Shanghai yuanye Bio-Technology Co., Ltd (Shanghai, China). LPS from *Escherichia coli* 055:B5 was obtained from Sigma (St. Louis, MO, USA). SIRT1 inhibitor EX-527 was obtained from Selleck Chemicals (Houston, TX, USA).

### In vivo study

#### Animal experiment

Health adult female C57BL/6 mice aged 8–12 weeks were obtained from the Beijing Vital River Company (Beijing, China). All mice were maintained at room temperature (22–24 °C) and fed with standard food and water under a 12:12 h light/dark cycle for 7 days before the experiments. The experiments were performed in accordance with Animal Ethics Committee of Huaihe Hospital of Henan University. The mice were randomly divided into four groups (10 in each group): sham group, LPS group, LPS + GA group, and LPS + GA + EX-527 group. The mice of LPS group were injected intraperitoneally (i.p.) with 10 mg/kg body weight of LPS (Sigma) to induce AKI. The mice in LPS + GA group were intraperitoneally administered with 200 mg/kg GA (Shanghai yuanye Bio-Technology Co., Ltd) 1 h before LPS challenge. The mice in LPS + GA + EX-527 group were further intraperitoneally administrated with 5 mg/kg EX-527 after LPS and GA treatment. In the sham group, the mice were injected with equal amount of PBS. The mice were euthanized by cervical dislocation at 24 h after LPS injection. The blood and urine samples were collected and kidney tissues were removed for measurement of renal function.

#### Renal function analysis

The blood and urine samples were collected and centrifuged at 4 °C for 20 min in glass tubes. The urine level of kidney injury molecule-1 (Kim-1) was measured by a highly sensitive two-site ELISA (ALPCO diagnostics, Salem, NH, USA). To assess renal function, the concentrations of blood urea nitrogen (BUN) in serum was analyzed by using commercial kit reagents (Institute of Jiancheng Bioengineering, Nanjing, China) using an Olympus AU2700 automatic biochemistry apparatus (Olympus America Inc., Melville, NY, USA).

#### Terminal deoxynucleotidyl transferase (TdT)-mediated dUTP-biotin nick end labeling (TUNEL) assay

Cell apoptosis in kidney sections was detected by TUNEL assay using an In Situ Cell Death Detection Kit (Roche Diagnostics, Indianapolis, IN). The number of TUNEL-positive nuclei was counted in six randomly fields of renal medulla under the Olympus BX53 microscope at a magnification of 400×. The apoptotic index was expressed as the percentage of TUNEL-positive cells of the total cell nuclei in each field.

#### Assay of caspase-3 activity

Protein from kidney tissues was used to determine caspase-3 activity. The activity of relative caspase-3 in kidney was conducted by using the caspase-3 activity kit (Bestbio, China) according to the manufacturer’s instruction.

### In vitro study

#### Cell culture and treatment

Human kidney epithelial cells (HK-2) were purchased from the National Platform of Experimental Cell Resources for Science (Beijing, China) and cultured in DMEM/F12 medium (Thermo Fisher Scientific, Pittsburgh, PA, USA) containing 10% fetal bovine serum (FBS; Invitrogen, Carlsbad, CA, USA), 1% penicillin–streptomycin (Gibco Life Technologies, Carlsbad, CA, USA) and 1% insulin–transferrin–selenium (Sigma) at 37 °C in a humidified chamber with 5% CO_2_. HK-2 cells were grown for 24 h to reach approximately 90% confluence before being randomly divided into three groups: control group, LPS group (LPS 10 μg/ml), LPS + GA group (LPS 10 μg/ml + GA 50 μg/ml). After 24 h treatment, the cells were harvested for various analyses. For LPS + GA + si-SIRT1 group, cells were transiently transfected with 50 nM si-SIRT1 (GenePharma, Shanghai, China) by Lipofectamine 2000 (Invitrogen) prior to LPS and GA treatment.

#### Quantitative real-time PCR (qRT-PCR)

Total RNA from normal human renal tissues and cultured HK-2 cells was isolated with TriReagent (Sigma). qRT-PCR analysis was carried out using SYBR Green PCR master mix (Applied Biosystems) on Bio-Rad iQ5 Multicolor Real-Time qRT-PCR Detection system (Bio-Rad, Hercules, CA, USA), in one-step RT-PCR protocol as previously described [21]. The relative expressions of IL-6, TNF-α and IκBα mRNA were normalized to GAPDH expression and examined by using the 2^−ΔΔCT^ method [22]. The primers used in this study were listed as follows: IL-6, forward 5′-ATGAACTCCTTCTCCACAAGCGC-3′, reverse 5′-GAAGAGCCCTCAGGCTGG ACTG-3′; TNF-α, forward 5′-CAGGGGCCACCACGCTCTTC-3′, reverse 5′-CTTGGGGCAGGGGCTCTTGAC-3′; IκBα, forward 5′-CGTGTCTGCACCTAGCCTCTATC-3′, 5′-GCGAAACCAGGTCAGGATTC-3′.

#### Western blot

Protein from collected kidney tissues and HK-2 cells were extracted using RIPA lysate (Thermo Fisher Scientific). The total protein concentrations were determined using the bicinchoninic acid (BCA) protein assay (Pierce, Rockford, IL, USA). Samples with equal amounts of protein were separated electrophoretically by 10% sodium dodecyl sulfate–polyacrylamide gel electrophoresis (SDS-PAGE) and electroblotted onto polyvinylidene difluoride (PVDF) membranes (Millipore, Billerica, MA, USA). Following blocked with 5% skim milk powder at room temperature for 2 h, the membranes were incubated with indicated antibodies including phosphorylated IκBα (p-IκBα), SIRT1, and inducible nitric oxide synthase (iNOS) (1:1000; Santa Cruz Biotechnologies, Santa Cruz, CA, USA) at 4 °C overnight. β-actin was used as a loading control. All the blots were subsequently incubated with horseradish peroxidase-conjugated anti-rabbit IgG antibody (Santa Cruz Biotechnology) for 1 h. Finally, the proteins were detected by an enhanced chemiluminescent detective system (Amersham Biosciences UK Ltd., Little Chalfont, UK).

#### ELISA analysis of TNF-α, IL-6 and NF-κB

The levels of TNF-α and IL-6 in plasma and supernatant of treated HK-2 cells and NF-κB in kidney homogenate were determined by using ELISA kits (R&D Systems Inc, Minneapolis, MN) according to the manufacturer’s instructions.

#### Statistical analysis

Data from each group were shown as the mean ± standard deviation (SD) from three independent experiments. All statistical analysis was performed using SPSS 16.0 software system (SPSS, Chicago, IL, USA). Comparison was determined by Student’s *t* test between two groups and one-way ANOVA tests among three or more groups. *P* value <0.05 was considered statistically significant.

## Results

### GA attenuated LPS-induced inflammatory response and renal injury

**Fig. 1:**
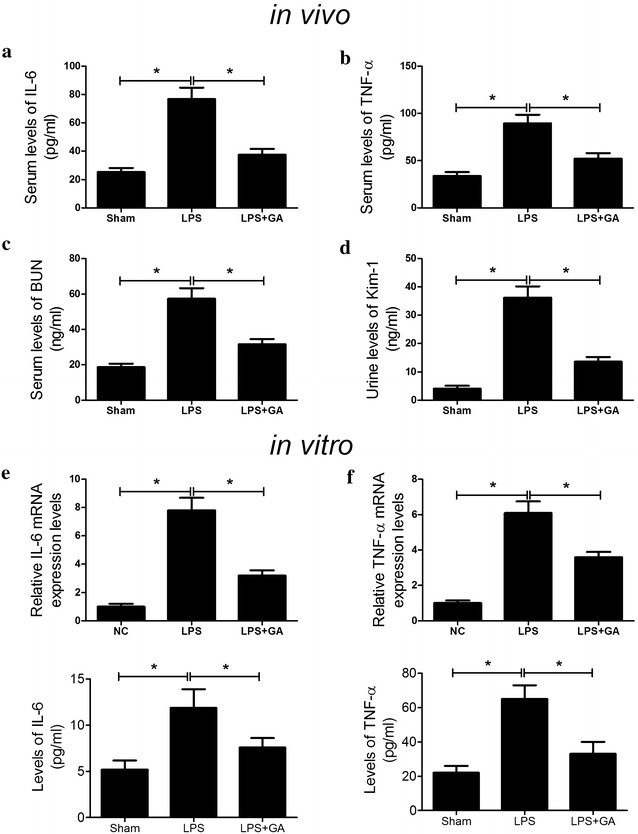
GA alleviated LPS-induced inflammatory response and renal injury. The levels of IL-6 (**a**) and TNF-α (**b**) in serum were quantified by ELISA at 24 h after LPS administration in sham, LPS and LPS + GA groups. The levels of BUN **c** in serum and Kim-1 **d** in urine were detected 24 h after LPS treatment in sham, LPS and LPS + GA groups. n = 10, *P < 0.05. qRT-PCR and ELISA was performed to detect the mRNA expressions and concentrations of IL-6 (**e**) and TNF-α (**f**) in HK-2 cells in control, LPS and LPS + GA groups. n = 3, *P < 0.05

To investigate the effect of GA on LPS-induced inflammatory response, the levels of inflammatory cytokine TNF-α and IL-6 induced by LPS in vivo and in vitro were determined. As demonstrated by ELISA kits, the serum concentrations of TNF-α and IL-6 in LPS-induced mice were both significantly increased at 24 h after LPS injection compared with sham group, whereas GA pretreatment dramatically reduced LPS-induced improvement of TNF-α and IL-6 levels (Fig. 1a, b). Consistently, the mRNA expressions and concentrations of TNF-α and IL-6 in HK-2 cells were evaluated and we found that LPS effectively improved the mRNA expressions and concentrations of TNF-α and IL-6 in HK-2 cells compared with control group, which were conspicuously inhibited by GA treatment (Fig. 1e, f). These results suggested that GA alleviated LPS-induced inflammatory response by reducing the production of inflammatory cytokines. As important indexes of renal injury severity, BUN and Kim-1 were utilized for the assessment of renal function. As illustrated in Fig. 1c, d, significantly elevated levels of BUN and Kim-1 were observed in the mice treated with LPS with respect to sham group. However, GA treatment markedly reduced the levels of BUN and Kim-1 induced by LPS, indicating that GA attenuated LPS-induced renal injury. Taken together, we concluded that GA challenge exerted a protective effect on renal function.

### GA inhibited renal cell apoptosis in LPS-induced AKT

**Fig. 2:**
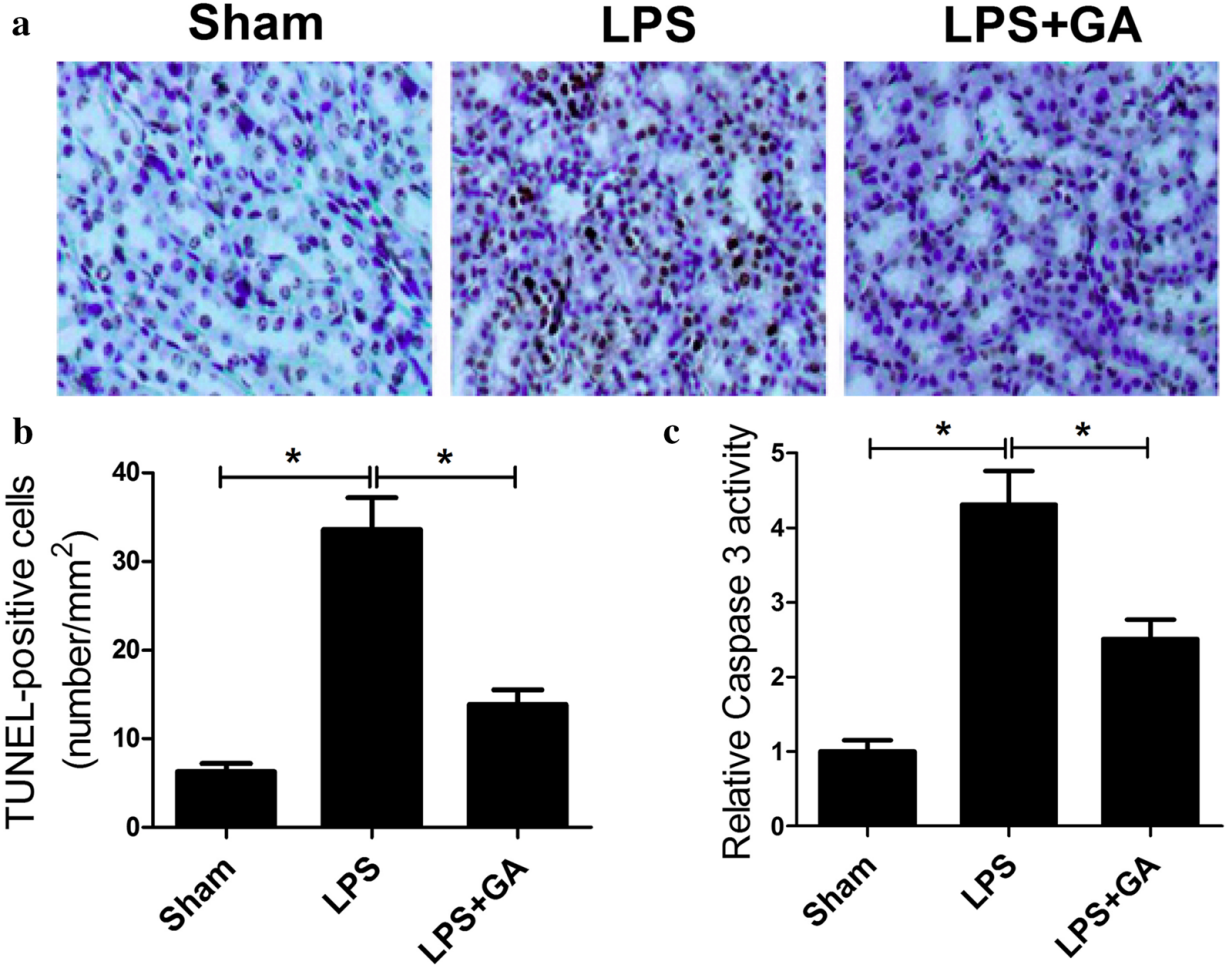
GA reduced cell apoptosis in LPS-induced AKT in vivo. **a** Representative photomicrographs of TUNEL staining in sham, LPS and LPS + GA groups. **b** Quantitative analysis of the number of apoptotic nuclei under TUNEL staining from six random fields in sham, LPS and LPS + GA groups. **c** Caspase-3 activities in sham, LPS and LPS + GA groups were detected using the caspase-3 activity kit. n = 10, *P < 0.05

To assess the effect of GA on renal tubular cell death in LPS-induced AKI, TUNEL staining was employed to determine the percentage of apoptotic cells in renal slides. As shown in Fig. 2a, b, a significant increase was observed in TUNEL-positive tubular cells in LPS group compared with sham group. However, GA pre-treatment strikingly reduced the number of LPS-induced TUNEL-positive cells. In agreement with TUNEL assay results, LPS administration led to a significant augment of casapse-3 activity in comparison with sham group, which was dramatically repressed by GA pre-treatment (Fig. 2c). These results demonstrated that GA effectively prevented LPS-induced renal cell apoptosis in the kidney.

### GA elevated the protein level of SIRT1 and restrained the NF-κB signaling pathway in LPS-induced AKT

**Fig. 3:**
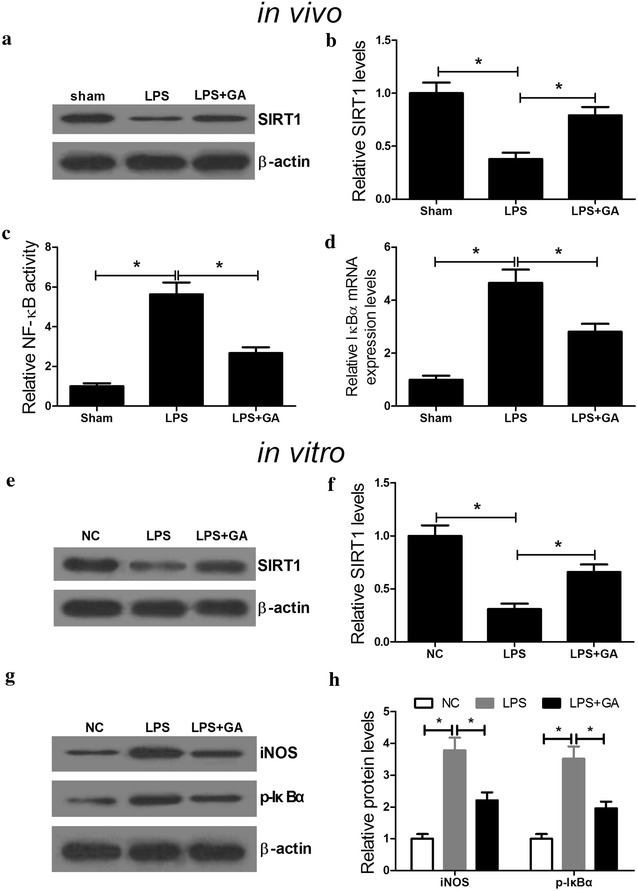
GA promoted the expression level of SIRT1 and suppressed the NF-κB signaling pathway. C57BL/6 mice and HK-2 cells were treated with LPS with or without GA. Western blot was performed to determine the expression levels of SIRT1 in renal tissues (**a**, **b**) and HK-2 cells (**e**, **f**). **c** The activity of NF-κB in renal tissues was measured by ELISA. **d** The protein levels of p-IκBα and IκBα in renal tissues was examined by western blot. n = 10, *P < 0.05. **g**, **h** The levels of iNOS, p-IκBα and IκBα in treated HK-2 cells were detected by western blot. n = 3, *P < 0.05

It was reported that Gingko biloba extract EGb761 could activate SIRT1 and prevent the activation of NF-κB against beta-amyloid-induced neurotoxicity, which was mediated through oxidative stress [23]. Thus, we analyzed the effect of GA on the expression of SIRT1 and the NF-κB signaling pathway. Western blot analysis showed that LPS markedly restrained the expression level of SIRT1 in renal tissues (Fig. 3a, b) and HK-2 cells (Fig. 3e, f) with respect to sham and control groups, but GA significantly overturned LPS-induced suppression on SIRT1 expression. Given the fact that IκBα, one of the key factors in the NF-κB pathway, regulates the transcription activity of NF-κB [24] and NF-κB is one of the well-known inducers of iNOS [25]. Therefore, to verify whether addition of GA triggered the inhibition of NF-κB signaling pathway, the activity of NF-κB and the expressions of IκBα and iNOS were assessed in vivo and in vitro. It is indicated that the relative activity of NF-κB was strikingly increased by LPS while combination treatment of GA and LPS dramatically inhibited LPS-induced NF-κB activity (Fig. 3c). Western blot results revealed that LPS markedly improved the ratio of p-IκBα/IκBα in renal tissues, whereas GA pretreatment partially abrogated this effect (Fig. 3d). As demonstrated by western blot, the levels of iNOS and p-IκBα were obviously boosted in LPS-induced HK-2 cells, however, GA pretreatment dramatically abolished this effect, suggesting that GA conspicuously downregulated iNOS and phosphorylation of IκBα (Fig. 3g, h). Taken together, these findings revealed that GA challenge upregulated the expression level of SIRT1 and inactivated the NF-κB signaling pathway.

### GA attenuated LPS-induced inflammatory response and renal injury by activating SIRT1 via inhibiting the NF-κB signaling pathway

**Fig. 4:**
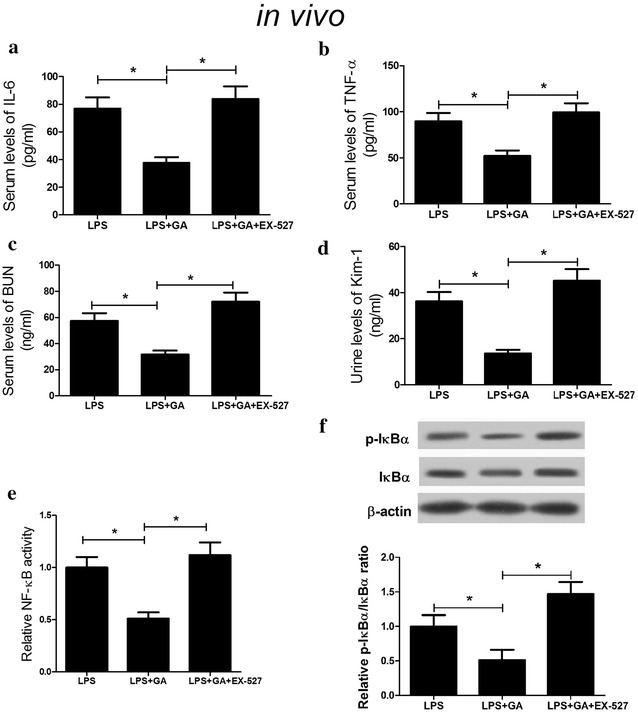
Suppression of SIRT1 reversed the effect of GA on LPS-induced inflammatory response and renal injury in vivo. The levels of IL-6 (**a**), TNF-α (**b**), BUN (**c**), Kim-1 (**d**), NF-κB activity (**e**) were measured in LPS, LPS + GA, and LPS + GA + EX-527 groups in vivo. **f** The protein levels of p-IκBα and IκBα in LPS, LPS + GA, and LPS + GA + EX-527 groups in vivo were determined by western blot. n = 10, *P < 0.05

**Fig. 5:**
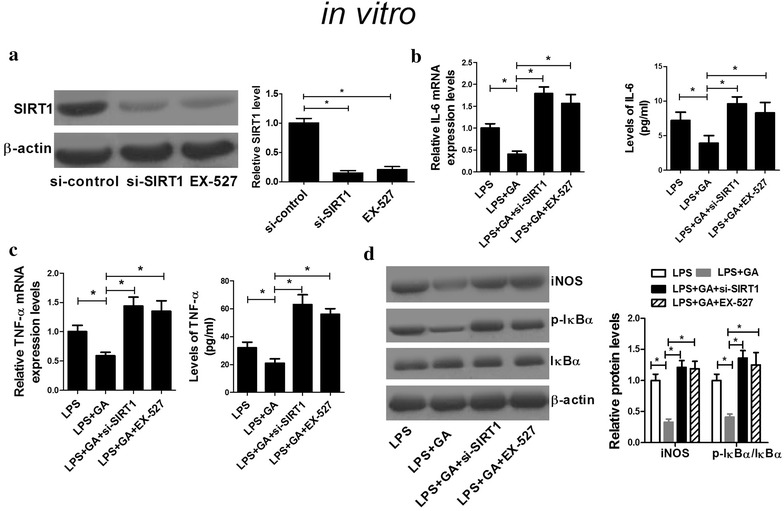
Suppression of SIRT1 reversed the effect of GA on LPS-induced inflammatory response and renal injury in vitro. **a** The protein level of SIRT1 in si-SIRT1, si-control, or EX-527 treated HK-2 cells in vitro was assessed by western blot. The mRNA expressions and concentrations of IL-6 (**b**) and TNF-α (**c**) in LPS, LPS + GA, LPS + GA + si-SIRT1, and LPS + GA +EX-527 groups were determined by qRT-PCR in vitro. **d** The levels of iNOS, p-IκBα and IκBα in LPS, LPS + GA, LPS + GA + si-SIRT1, and LPS + GA +EX-527 groups were detected by western blot in HK-2 cells. n = 3, *P < 0.05

We further explored the underlying mechanism by which GA exerted a protective role in LPS-induced AKI. EX-527, an inhibitor of SIRT1, was used to explore whether GA exerted a renoprotective role in vivo by regulating SIRT1. ELISA results demonstrated that the serum levels of inflammatory cytokines TNF-α and IL-6 in mice were significantly decreased in LPS + GA group while EX-527 administration markedly abolished this effect (Fig. 4a, b). Additionally, GA led to a marked reduction in BUN and Kim-1 induced by LPS in mice while EX-527 treatment obviously reversed this effect (Fig. 4c, d). Furthermore, EX-527 treatment also relieved (LPS + GA)-induced suppression on NF-κB activity (Fig. 4e) and the ratio of p-IκBα/IκBα (Fig. 4f) in renal tissues of mice. si-SIRT1 was employed to further investigate the mechanism of GA function in vitro. Western blot analysis demonstrated that si-SIRT1 transfection and EX-527 treatment both significantly reduced the protein level of SIRT1 (Fig. 5a). As expected, si-SIRT1 transfection and EX-527 treatment both significantly restored (LPS + GA)-induced decrease of mRNA expression and concentrations of inflammatory cytokines IL-6 (Fig. 5b) and TNF-α (Fig. 5c). Furthermore, si-SIRT1 administration and EX-527 treatment both dramatically recuperated (LPS + GA)-induced repression on the levels of iNOS and phosphorylation of IκBα (Fig. 5d). Taken together, these results indicated that GA attenuated LPS-induced inflammatory response and renal injury by activating SIRT1 via inhibiting the NF-κB signaling pathway.

## Discussion

AKI, associated with several complications, is well known as a clinical entity with rapid decline in renal function, contributing to multiorgan failure in critically ill patients [26]. Considering the significant acute morbidity and mortality of septic AKI, novel therapeutic medical is urgently needed to treat or prevent AKI.

EGB has been reported to be widely used for the treatment of cardiovascular disorders, diabetes mellitus, and various types of cancers due to its various biological activities like antioxidant, anti-inflammatory, anti-proliferative, and anti-tumorigenic effects [27]. Previous studies have reported that GBEs play a protective effect on renal injury. For example, Nazarenko et al. reported that Bilobil, a commercial preparation based on *Ginkgo biloba* extract, exerted a nephroprotective effect on experimental AKI by simulating the renal antioxidant system, decreasing the rate of lipid peroxidation processes and reducing the renal damage [28]. Akdere et al. found that *Ginkgo biloba* EGb761 extract protected rats from renal ischemia–reperfusion injury [29]. Sener et al. demonstrated that EGB ameliorated ischemia reperfusion-induced renal injury in rats probably by the radical scavenging and antioxidant activities [30]. In the present study, we successfully constructed a LPS-induced murine AKI model to firstly assess the protective effects of a novel GBE GA. We found that GA administration protected mice from LPS-induced AKI by attenuating LPS-induced inflammatory response, renal injury, as well as tubular apoptosis both in vivo and in vitro.

Increasing evidence has suggested that SIRT1 is implicated in renal physiology and pathology, and plays a renal protective role against inflammatory injury [31]. For example, Xu et al. reported that SIRT1/3 activation by resveratrol attenuated AKI following sepsis in a septic rat model [32]. Wang et al. uncovered that resveratrol, a SIRT1 activator, ameliorated the renal tubular injury induced by hyperglycemia both in vitro and in vivo via suppressing apoptosis [33]. NF-κB is a important transcription factor that regulates inflammatory reaction by inducing gene expression of inflammatory cytokines, chemokines, as well as inflammatory enzymes (iNOS) [34]. Notably, it is stated that NF-κB has been considered as a central link in the pathogenic processes of inflammatory reaction and renal injury induced by LPS [35]. Resolvin D1 protected LPS-induced AKI by downregulating NF-κB inflammatory signal as well as inhibiting renal cell apoptosis [36]. Leonurine reduced kidney injury and protected renal functions from LPS-induced AKI by inhibiting ROS-mediated NF-κB signaling pathway [37]. In our present study, we found that LPS significantly reduced SIRT1 expression and activated the NF-κB signaling pathway, consistently with these previous studies. Further mechanistic analyses revealed that GA exerted its renoprotective role in LPS-induced AKI by activating SIRT1 via inhibiting the NF-κB signaling pathway.

## Conclusions

In conclusion, the present study demonstrated that GA administration protected mice from LPS-induced AKI by attenuating LPS-induced inflammatory response, renal injury, as well as tubular apoptosis. GA also suppressed inflammatory response induced by LPS in HK-2 cells. GA exerted renoprotective role in LPS-induced AKI by activating SIRT1 via inhibiting the NF-κB signaling pathway. Overall, our study provides evidence for the role of GA as a promising therapeutic agent for septic AKI.

## Abbreviations

GA: ginkgetin aglycone
GBE: *Ginkgo biloba* extract
AKI: acute kidney injury
LPS: lipopolysaccharide
TLR-4: toll-like receptor-4
TNF-α: tumor necrosis factor-α
NF-κB: nuclear factor-κB
ROS: reactive oxidative species
ELISA: enzyme-linked immunosorbent assay
Kim-1: kidney injury molecule-1
BUN: blood urea nitrogen
TdT: terminal deoxynucleotidyl transferase
qRT-PCR: quantitative real-time PCR
BCA: bicinchoninic acid
SDS-PAGE: sodium dodecyl sulfate–polyacrylamide gel electrophoresis
p-IκBα: phosphorylated IκBα
iNOS: inducible nitric oxide synthase
