# Editorial Expression of Concern: Ginkgetin aglycone ameliorates LPS-induced acute kidney injury by activating SIRT1 via inhibiting the NF-κB signaling pathway

**DOI:** 10.1186/s13578-022-00837-z

**Published:** 2022-06-30

**Authors:** Junwei Zhang, Suxia Yang, Fang Chen, Huicong Li, Baoping Chen

**Affiliations:** Department of Nephrology, Huaihe Hospital of Henan University, No. 115, Gulou District, Kaifeng, 475000 China

## Correction to: Cell Biosci (2017) 7: 44 https://doi.org/10.1186/s13578-017-0173-3

The Editors-in-Chief would like to alert readers that concerns have been raised regarding the data in this article [1]. Specifically, there appear to be inconsistencies in the western blot image backgrounds in Figs. 3a, 3 g and 4f. Additional concerns have been raised by readers regarding Fig. 2a, where repetitive cell patterns have been observed. Furthermore, the article doesn't provide sufficient detail about ethics approval. Therefore, readers are advised to interpret the results with caution.

None of the authors have responded to any correspondence from the editor or publisher about this Editorial Expression of Concern.
